# Experimental Evaluation of the Influence of Aggregate Strength on the Flexural Cracking Behavior of Epoxy Asphalt Mixtures

**DOI:** 10.3390/ma13081876

**Published:** 2020-04-16

**Authors:** Wei Xu, Xiaoshu Wei, Jintao Wei, Zhengxiong Chen

**Affiliations:** School of Civil Engineering and Transportation, South China University of Technology, Wushan Road, Tianhe District, Guangzhou 510641, China; 201721008591@mail.scut.edu.cn (X.W.); ctweijintao@mail.scut.edu.cn (J.W.); 201920107290@mail.scut.edu.cn (Z.C.)

**Keywords:** epoxy asphalt mixture, aggregate strength, flexural cracking, impact toughness, experimental study, bending test, fatigue test

## Abstract

The flexural cracking resistance of an asphalt concrete mixture used in a steel bridge deck pavement needs to be higher than that of one used in ordinary pavement. In this study, mechanical experimental tests were used to evaluate the influence of the aggregate strength on the flexural cracking behavior of epoxy asphalt concrete (EAC). The aggregate fracture area of beam cross sections was quantitatively analyzed by digital image processing, and crack propagation in the mixture was analyzed using fracture mechanics theory. The bending test results showed that the EAC containing high-strength aggregates exhibited the highest flexural cracking resistance among all of the aggregate mixtures under the same conditions. The use of high-strength aggregates led to a reduction in the aggregate fracture area, thereby improving the flexural cracking resistance of the mixture. The aggregate strength had a significant influence on the flexural cracking propagation behavior of the mixture. Fatigue test results at strain-controlled levels of 600–1200 με and 15 °C showed that the aggregate strength had no evident influence on the fatigue properties of the EAC. It is recommended that high-strength aggregates are used to increase the fracture resistance of aggregates and the flexural crack resistance of EACs.

## 1. Introduction

Epoxy asphalt concrete (EAC) is composed of asphalt, epoxy resin, a curing agent, and aggregates, and is characterized by good waterproofing performance, rutting resistance, high strength, and fatigue resistance. EAC is widely used in orthotropic steel bridge deck pavement engineering [1,2]. The steel plates of a steel bridge exhibit larger deformation under traffic loading than a concrete bridge deck or ordinary pavement. Hence, a steel bridge deck pavement should have good deformation compatibility, which requires an EAC with high deformation resistance. The traffic load causes flexural deformation in the steel bridge deck pavement, generating large shear and tensile stresses in the pavement that make the mixture prone to fatigue cracking [3,4]. After cracks appear in the pavement, water can enter the interior of the pavement along the cracks and accelerate pavement damage. Therefore, it is very important to study the factors that influence EAC flexural cracking behavior.

Mineral materials, which include aggregates and fillers comprising over 90% of the total mass of an asphalt mixture, significantly affect the mixture performance. Aggregates interlock to form a supporting skeletal structure in an asphalt mixture. Fillers are mixed with asphalt into a mastic that fills in the skeletal voids, which is the key to ensuring the cohesion of the mixture [5,6]. Kollmann et al. [7] used a cohesive zone model to simulate the initiation and propagation of microcracks in an asphalt mixture in an indirect tensile test. The fracture failure of the asphalt mixture was found to result from the continuous development and expansion of microcracks and microvoids in the concrete mixture under loading. The existence of air voids results in the development of more distinct stress concentrations, which in turn, lead to reduced load-bearing capacities. The damage distribution showed that the aggregate–mortar interface exhibited more significant damage than the bulk fracture in the mortar itself. As the temperature increases, the ductility of the asphalt mortar increases. The mixture showed pronounced deformation before damage, and the damage distribution was more uniform. Low temperatures such as −10 °C were associated with significant localized damage in the mixture due to brittle fracture behavior. Inside the asphalt mixture, the interfacial bond strength between the aggregates and the asphalt is an important factor in the flexural cracking behavior of the asphalt mixture. Studies have shown that using angular aggregates with a rich surface texture and high roughness can enhance the interfacial bond strength between the aggregate and the asphalt, which in turn significantly improves the fatigue cracking resistance of the asphalt mixture [8,9,10]. Haskett et al. [11] analyzed the shear friction and interlocking behavior of aggregates. The mineral material skeleton formed by the interlocking of coarse and fine aggregates was found to be conducive to improving the internal friction and load-bearing capacity of the material, thereby increasing the resistance to bending and shear stresses from traffic loading. Golalipour et al. [12] showed that improving the gradation design and reducing the air void percentage and number of voids in a mineral aggregate enhanced the rutting resistance of an asphalt mixture. 

The aggregate strength characteristics also affect the flexural cracking behavior of asphalt mixtures. Moreno and Rubio [13] carried out an asphalt fatigue cracking test (UGR-FACT) to evaluate the fatigue cracking behavior of a diabase asphalt mixture and a limestone mixture. The test results showed that the diabase aggregates did not fracture because of a high crushing strength, and the corresponding asphalt mixture had a higher resistance to fatigue cracking. In comparison, the limestone asphalt mixture exhibited a few aggregate fractures and a short fatigue life, indicating that high-strength aggregates provide higher resistance against crack propagation. Mahmoud [14] integrated the discrete element method with image processing technology to model and analyze the modulus, compressive strength, and indirect tensile strength of five aggregates including granite, hard limestone, soft limestone, gravel, and sandstone. The effect of the aggregate strength on the fracture property of the asphalt mixture was evaluated, and the results showed that the crushing of coarse aggregates reduced the internal force between particles. Thus, the mechanical properties and the load-bearing capacity of the asphalt mixture were reduced. Aliha et al. [15] investigated the influence of limestone and siliceous aggregates on the fracture performance of an asphalt mixture using a semicircular bending (SCB) test. The limestone aggregates had a higher strength and stiffness and a correspondingly higher fracture resistance than the siliceous aggregates. Li and Marasteanu [16] conducted an SCB fracture test to compare the influence of using granite and limestone on the fracturing of an asphalt mixture. Granite is more resistant to crushing than limestone. The test results showed that while a significant portion of the fracture passed through the aggregate particles in the mixture made with limestone, most of the fracture in the mixture made with granite passed through the interface with the mastic. Under the same low-temperature conditions, the granite asphalt mixture had a higher peak load and fracture energy and exhibited higher fracture resistance than the limestone asphalt mixture.

In addition to the shape and surface roughness of the aggregates affecting the performance of the asphalt mixture, the mechanical properties of the aggregates also influenced the mixture cracking resistance, which has attracted the attention of researchers. Vishalakshia et al. [17] performed tests to comparatively evaluate the strength and cracking behavior of ordinary and high-strength concrete containing five different types of aggregates. The aggregate type was found to strongly influence the strength of high-strength concrete, but had no significant influence on ordinary-strength concrete. Generally, asphalt mixtures, especially epoxy asphalt mixtures, exhibit clear elastic characteristics and higher flexural strengths at low temperatures, similar to cement concrete. The influence of the relative strength of aggregate strength and mastic strength of cement concrete on cracking characteristics has reference significance. Ipek [18] evaluated the effect of artificial aggregates on the mechanical properties, fracture parameters, and bond strength of cement concrete. The mechanical and fracture properties of concretes with similar strengths were significantly affected by the incorporation of artificial lightweight aggregates. Thus, as the concrete strength increases, the influence of aggregates becomes increasingly significant.

A crosslinking reaction between epoxy resin and asphalt can be used to create a three-dimensional network. The high cohesion and strength of epoxy asphalt significantly improve the properties of EAC including the strength and viscoelasticity over those of ordinary asphalt mixtures [19]. In the literature, EACs have been designed and evaluated mainly considering the properties of an ordinary asphalt mixture. Little attention has been paid to the effects of aggregate strength characteristics on the EAC fracture resistance or the corresponding correlation.

Considering the influence of aggregates on the properties of ordinary asphalt mixtures and cement concrete, aggregates are expected to significantly affect the EAC properties. The effects of aggregate properties on the EAC mechanical properties were evaluated in this study by comparative tests on three types of EACs containing aggregates of different strengths. First, a Marshall stability test was carried out, followed by a three-point bending test to evaluate the flexural tension resistance and impact toughness of the three types of EACs. Finally, a single-edge notched beam bending test was used to evaluate the fracture toughness of three types of EACs. After the beam bending tests, data from the specimen fracture cross sections were collected, the aggregate fracture area was statistically quantified, and the fracture morphology of the aggregate particles was analyzed to comprehensively evaluate the effect of aggregate strength on EAC flexural cracking behavior. To evaluate the effects of cyclic loading, four-point bending fatigue tests were conducted on three types of EACs.

## 2. Materials and Methods

### 2.1. Materials

#### 2.1.1. Asphalt Binder

Three types of asphalt binder were tested including epoxy asphalt, base asphalt, and styrene-butadiene-styrene (SBS) -modified asphalt. The epoxy asphalt was a 50:50 mass ratio mixture of KD-BEP epoxy resin (Kindai Kasei Corp., Ltd., Aichi-gun, Aichi-ken, Japan) and A-70 base asphalt (Shell Xinyue (Foshan) Asphalt Co. Ltd., Foshan, Guangdong, China) [20]. Table 1, Table 2 and Table 3 show the performance indexes of the A-70 base asphalt, epoxy asphalt, and SBS asphalt (Shell Xinyue (Foshan) Asphalt Co. Ltd., Foshan, Guangdong, China).

#### 2.1.2. Aggregates and Mineral Fillers

**Figure 1:**
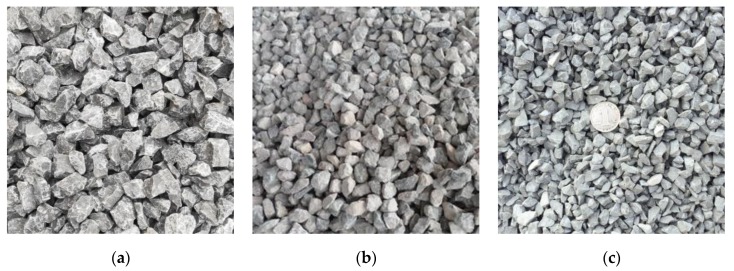
The coarse aggregate sample: (**a**) diabase, (**b**) granite, and (**c**) limestone.

The effects of different aggregate strengths on the EAC mechanical properties were compared using three types of aggregates, namely, diabase, granite, and limestone in the tests. Pictures of the three types of coarse aggregates are shown in Figure 1. The technical indexes of the coarse aggregates are shown in Table 4. The chemical compositions of the three types of coarse aggregates are shown in Table 5. The test results showed different compressive strengths for the three types of aggregates. The morphological characteristics of aggregates can affect the mechanical properties of the mixture. All three types of aggregates were obtained from the same aggregate plant and underwent the same crushing production process. The technical indexes of the limestone powders that were used are shown in Table 6. An A-70 base asphalt mixture, SBS-modified asphalt mixture, and an epoxy asphalt mixture were compared using the diabase aggregates.

### 2.2. Gradation Design of the Mixture

The same gradation design was adopted with the three aggregate types. The Marshall test was used to determine the optimum asphalt–aggregate ratio. The air voids were controlled at the same level of approximately 1.0%. The mixture gradation is shown in Table 7.

## 3. Methods

### 3.1. Marshall Stability Test

The Marshall stability test is used to characterize the compression resistance and deformation properties of an asphalt mixture at high temperatures. This test empirically reflects the compressive load-bearing capability of EACs with different stone aggregates and was used to comprehensively evaluate the internal cohesion of the mixture and the shear friction of the aggregates. In the experiment, four specimens were tested for each type of EAC.

### 3.2. Stone Beam Bending Test

**Figure 2:**
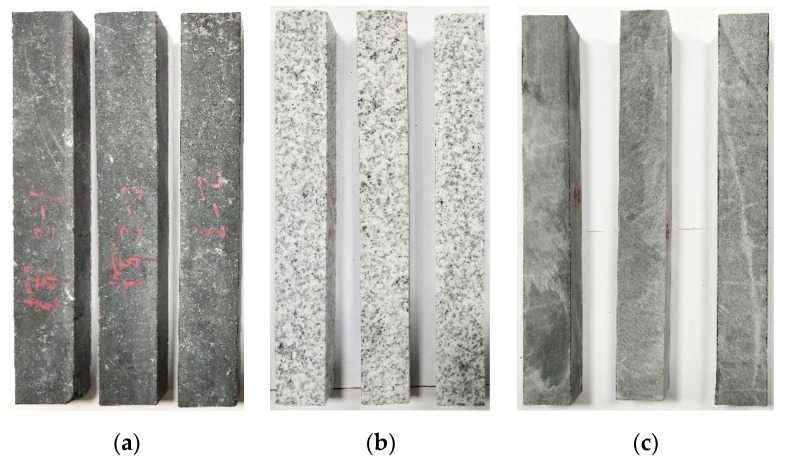
Three types of stone beam specimens: (**a**) diabase, (**b**) granite, and (**c**) limestone.

An epoxy asphalt pavement is subjected to relatively high bending stress; consequently, high flexural tensile strengths are required for the aggregates inside the mixture. Aggregate strength indexes include the compressive strength and the crushing value. As aggregate fracture failure is mainly determined by the flexural tensile properties, the flexural tensile strength of aggregate source stone has considerable engineering significance [21,22]. A three-point bending test was carried out on the aggregate source stone to determine the flexural tensile strength characteristics of the different aggregate types. Three types of raw stone materials, diabase, granite, and limestone, were cut into prismatic beam specimens with dimensions of 250 mm (length) × 30 mm (width) × 35 mm (height), as shown in Figure 2. The three-point stone beam bending test was conducted according to “Standard Test Methods of Bituminous Mixtures for Highway Engineering” (JTG E20-T0715-2011) using a MTS 810 material test system (MTS Systems Co., Eden Prairie, MN, USA) at a test temperature of 15 °C and a loading rate of 50 mm/min. In the experiment, six specimens were tested for each type of stone.

### 3.3. Three-Point Bending Test

**Figure 3:**
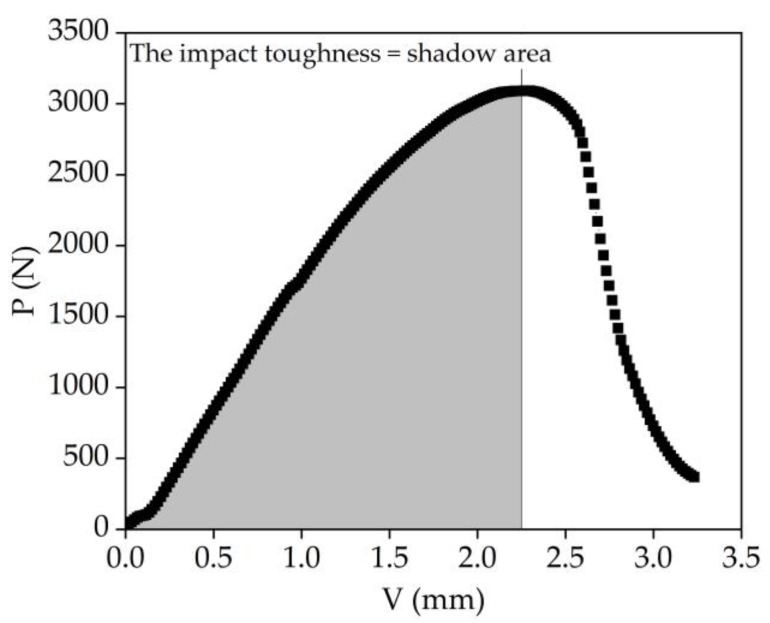
Calculation diagram of impact toughness by the area integral method.

**Figure 4:**
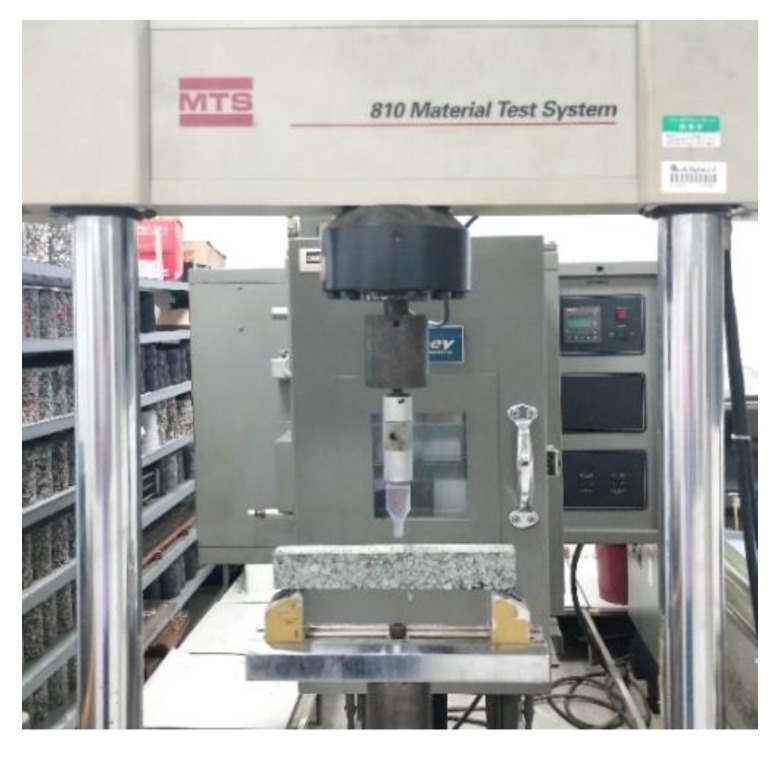
Three-point bending test device for the epoxy asphalt mixture beams.

**Figure 5:**
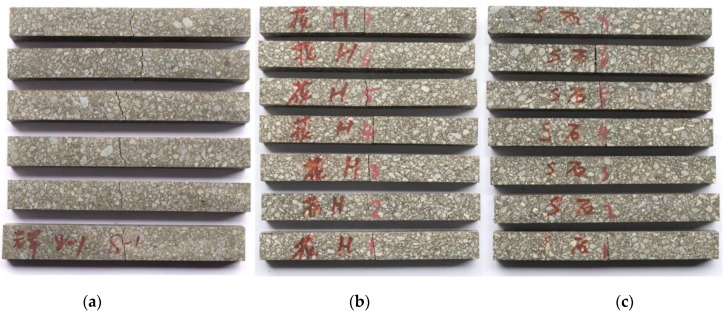
Three types of EAC beam specimens: (**a**) diabase, (**b**) granite, and (**c**) limestone.

A three-point bending test was conducted to evaluate the effect of the three aggregate types on the EAC flexural cracking behavior. Under continuous loading of the beam specimen, cracks formed and developed inside the mixture, eventually resulting in specimen fracture. The impact toughness (*A_K_*) of a material reflects its ability to resist deformation and fracturing under an impact load. Energy is dissipated from the fracturing of a beam specimen under the impact load. The load–displacement curve of the test data was used to calculate the EAC impact toughness by the area integral method using Origin software (Origin 2018, OriginLab, Northampton, MA, USA). The area under the loading portion of the load deflection curves, up to the maximum load, was measured from the curves presented in Figure 3. Zhang [23] conducted three-point and four-point bending tests and determined a good positive correlation between *A_K_* and fatigue performance in an EAC. In the present study, EACs with three types of aggregates were formed by the slab roller compacting method. After curing, the EACs were cut into beam specimens with dimensions of 250 mm in length, 30 mm in width, and 35 mm in height. The fracture property differences among the EACs, the base asphalt mixture, and the SBS asphalt mixture with the same diabase aggregate were investigated. The tests were carried out according to the “Standard Test Methods of Bituminous Mixtures for Highway Engineering” (JTG E20-T0715—2011) at test temperatures of 15 °C and −10 °C and a loading rate of 50 mm/min. The test device is shown in Figure 4. In the experiments, six specimens were tested for each mixture, as shown in Figure 5.

### 3.4. Single-Edged Bending of Mixtures

The fracture toughness of a mixture characterizes its ability to withstand crack propagation and is an indicator of the fracture performance of the mixture. Studies on asphalt mixtures have shown that the crack resistance increases with the fracture toughness [24,25,26]. The fracture toughness of asphalt mixtures is significantly influenced by the material composition characteristics and is commonly characterized by the fracture toughness index (*K_IC_*), which can be determined by a three-point bending test with a single-edge notched beam [27]. The maximum load *P_max_* is obtained experimentally and used to calculate *K_IC_* using the following formula recommended by the American Society for Testing Materials (ASTM):

(1) $$K_{IC}=\frac{P_{\max}S}{bh^{3/2}}f[\frac{a_{0}}{h}],$$

(2) $$f[\frac{a_{0}}{h}]=2.9{\left[\frac{a_{0}}{h}\right]}^{1/2}-4.6{\left[\frac{a_{0}}{h}\right]}^{3/2}+21.8{\left[\frac{a_{0}}{h}\right]}^{5/2}-37.6{\left[\frac{a_{0}}{h}\right]}^{7/2}+38.7{\left[\frac{a_{0}}{h}\right]}^{9/2},$$

where *S* is the specimen span (*S* = 200 mm); $a_{0\;}$is the length of the prefabricated notch; *h* is the specimen height; and *b* is the specimen width.

The beam specimens of the different EACs were 250 mm in length, 30 mm in width, and 35 mm in height. The prefabricated notch had a depth of 5 mm in the height direction and a width of 1 mm, so that the ratio of the notch depth to the specimen height *(a_0_/h*) was approximately 0.143. The test was carried out at −10 °C and 15 °C at a loading rate of 1 mm/min. In the experiment, six specimens were tested for each EAC.

### 3.5. Digital Image Processing

**Figure 6:**
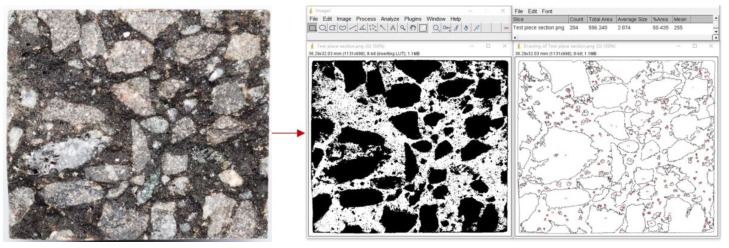
Beam fracture section digital image information acquisition.

The cross section of the asphalt mixture beam after flexural fracture contained voids, asphalt mastic, and aggregate. The aggregate fracture was related to the EAC fatigue cracking performance using digital image processing (DIP) to statistically quantify the aggregate fracture area on the specimen cross section. ImageJ is a Java-based image processing software developed by the National Institutes of Health (Version: 1.52q, NIH, Bethesda, MD, USA). The scale and units are set, and the image is processed using binarization, enhancement, and contour feature extraction. Then, the number of aggregates on the cross section is automatically extracted, and the fracture area of aggregates is statistically quantified, as shown in Figure 6. A high-definition digital camera was used for image acquisition, and the contour of the binarized image was enhanced using Photoshop software (Photoshop CC 2017, Adobe Systems, Inc., San Jose, CA, USA). In the experiment, six specimens were tested for each EAC.

### 3.6. Four-Point Bending Fatigue Test

**Figure 7:**
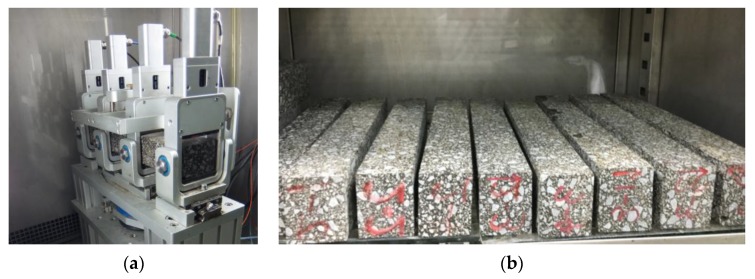
The test device and beam specimens: (**a**) four-point bending fatigue device and (**b**) EAC beam specimens.

Fatigue tests were conducted to understand the influence of cyclic loading on the flexural cracking behavior of epoxy asphalt mixtures on a steel bridge deck pavement. EAC samples were subjected to a four-point bending fatigue test assessed according to the American Association of State Highway Transportation Officials (AASHTO) T 321-03 at a temperature of 15 °C and a loading frequency of 10 Hz in the strain control mode using a NU-14 fatigue testing machine (Cooper Research Technology Ltd., Ripley, Derbyshire, UK). The failure point is defined as the point in the load cycle at which the specimen exhibits a 50% reduction in stiffness relative to the initial stiffness. The beam specimens used for the fatigue test were characterized by dimensions of 380 mm (length) × 63.5 mm (width) × 50 mm (height) and were obtained by cutting 400 × 300 × 70 mm slabs prepared using a laboratory roller compactor. The test device and beam specimens are shown in Figure 7. In the experiment, four specimens were tested for each EAC.

The EAC mixture sample exhibited an endurance limit at a strain level lower than 500 με, and its modulus basically became steady upon reaching 60–70% of the initial stiffness, after which there was no significant change in its fatigue trend. On this basis, the fatigue property differences among the EAC samples containing diabase, granite, and limestone were evaluated at strain levels of 600 με, 900 με, and 1200 με. The influences of aggregate strength on the fatigue and rheological properties of the EACs were analyzed.

The initial stiffness and phase angle of EACs were tested at the 50^th^ load cycle in the four-point bending test according to AASHTO T 321-03, at a loading frequency of 10 Hz in the strain of 600 με, and at temperatures of 0 °C, 15 °C, 30 °C, 45 °C, and 60 °C, respectively. The specimens were cured four hours before each temperature test.

## 4. Results

### 4.1. Flexural Tensile Properties of Stone

**Figure 8:**
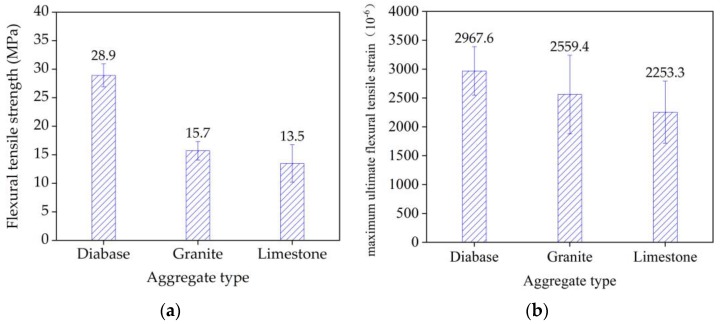
Three-point bending test results of three types of stone beams: (**a**) flexural tensile strength and (**b**) flexural tensile strain.

The test results for the aggregate property indexes are shown in Table 4. The three aggregate types had significantly different crushing values and compressive strengths in the following descending order: diabase > granite > limestone. The bending test results for the three stone materials are shown in Figure 8. The ultimate flexural tensile strains of the three types of stone are as follows, in descending order: diabase > granite > limestone. The compressive strength indexes of the three aggregate types were significantly correlated with the experimentally obtained flexural tensile properties of the stone; that is, stone materials with a high crushing resistance have a high flexural tensile strength.

### 4.2. Marshall Stability of the Mixture

The air void content, Marshall stability, and flow value are important indexes for an asphalt mixture. Similar gradation and air voids were used for the three EACs. The Marshall stability test results for the mixtures are shown in Table 8. There were no significant differences in the Marshall stabilities and flow values among the three types of mixtures, indicating similar resistances to compressive loading. The Marshall stability at 60 °C mainly resulted from combined aggregate interlocking, friction, and binder cohesion. Under loading, the aggregates did not reach their compressive strengths at failure, and the aggregate strength was not reflected in the Marshall test.

### 4.3. Three-Point Bending Test Analysis of Epoxy Asphalt, Base Asphalt, and SBS Modified Asphalt Mixture

**Figure 9:**
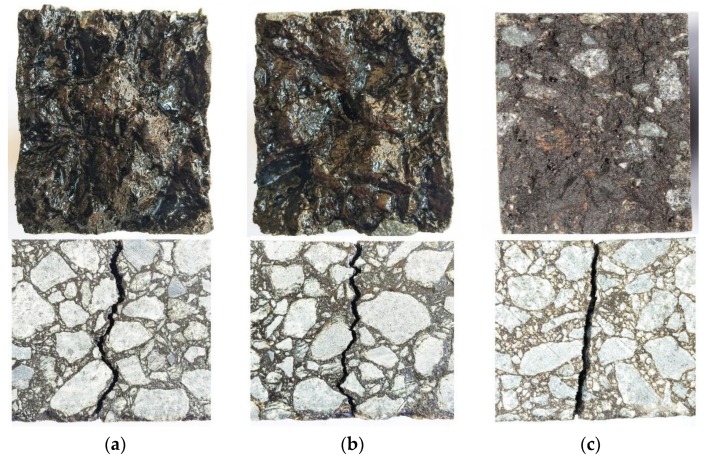
Beam fracture sections of different types of asphalt mixtures: (**a**) A-70, (**b**) SBS-modified asphalt, and (**c**) epoxy asphalt.

Three-point bending tests were carried out on an A-70 base asphalt mixture, an SBS-modified asphalt mixture, and an epoxy asphalt mixture under the same test conditions using the diabase aggregates. The optimum asphalt content of these asphalt mixtures was 6.5%. The test results are shown in Table 9. The EAC had a higher strength and higher maximum ultimate flexural tensile strain than the A-70 and SBS-modified asphalt mixtures. At 15 °C, compared with the values of the A-70 base asphalt mixture and SBS-modified asphalt mixture, the flexural tensile strength of the EAC increased by 218% and 199%, respectively, and the maximum ultimate flexural tensile strain of the EAC increased by 21% and 11%, respectively. At the test temperature of −10 °C, compared with the values of the A-70 base asphalt mixture and SBS-modified asphalt mixture, the flexural tensile strength of the EAC increased by 281% and 222%, respectively, and the maximum ultimate flexural tensile strain of the EAC increased by 96% and 31%, respectively. The EAC showed better low-temperature cracking resistance than the base asphalt mixture and SBS-modified asphalt mixture. Based on engineering application performance investigations of EAC on steel bridge decks for more than 12 years, low-temperature cracking issues are rare. A comparison of the cross sections of the different mixtures showed that there was basically no aggregate fracturing in the cross sections of the A-70 and SBS-modified asphalt mixtures and significant aggregate fracturing in the EAC cross section, as shown in Figure 9.

### 4.4. Flexural Tensile Properties of the Mixture

**Figure 10:**
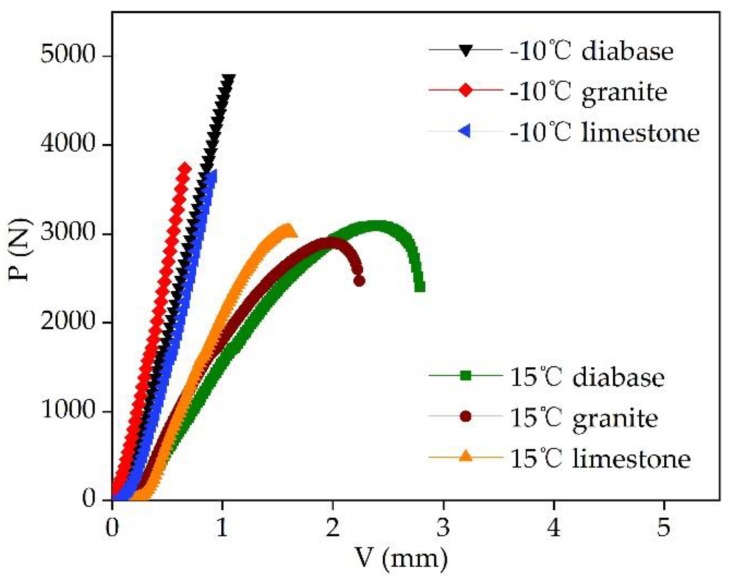
Load–displacement curves for the different EACs.

**Figure 11:**
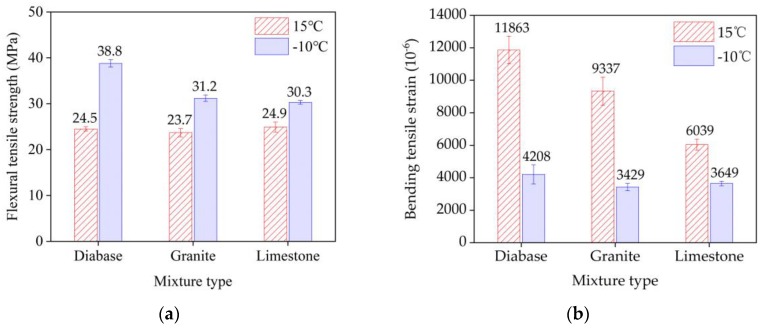
Three-point bending test results of three EAC beam types: (**a**) flexural tensile strength and (**b**) ultimate flexural tensile strain.

The three-point bending test results for EACs are shown in Table 10. The mid-span deflection and critical load of the three mixtures were not significantly different at 15 °C. Under the condition of −10 °C, the °C, diabase EAC exhibited the highest mid-span deflection and critical load, with values of 0.76 mm and 4618.4 N4N, respectively. The EAC load–displacement (P–V) curves at two temperatures are shown in Figure 10. The three types of EACs exhibited two fracture modes (i.e., elastoplastic fracturing at 15 °C and brittle fracturing at −10 °C). The EAC beam bending test results for the three aggregate types at these two temperatures are shown in Figure 11. The three aggregate mixtures exhibited significantly different tensile strengths at −10 °C and significantly different flexural tensile strains at 15 °C. The diabase EAC exhibited the highest flexural tensile strength and ultimate flexural tensile strain at the two temperatures; that is, the EAC with the high-strength aggregate had the highest flexural tensile resistance.

### 4.5. Impact Toughness and Fracture Toughness

**Figure 12:**
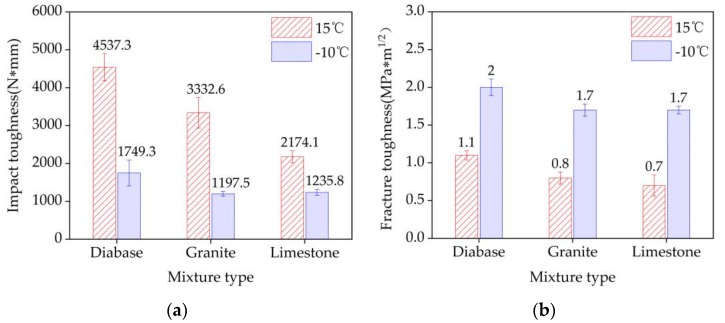
Three-point bending test results of the three mixture beam types: (**a**) impact toughness and (**b**) fracture toughness.

The *A_K_* and *K_IC_* values of the three EACs are shown in Figure 12. The mixture *A_K_* increased with the temperature, whereas *K_IC_* increased as the temperature decreased. Thus, the temperature significantly influenced the *A_K_* and *K_IC_* values of the mixture. The *A_K_* value characterizes the ability of a mixture to resist flexural deformation, and the *K_IC_* value characterizes the resistance of the mixture to cracking. The diabase EAC had relatively high *A_K_* and *K_IC_* values at the two temperatures, indicating that the use of high-strength aggregates significantly improved the EAC flexural cracking resistance.

### 4.6. Aggregate Fracture Area of Flexural Specimen

There were many aggregate fractures in the cross section of the EAC beam specimen. ImageJ software was used to statistically calculate the aggregate fracture area. The aggregate fracture area in the cross section of the different specimens was compared in terms of the aggregate fracture area ratio:

(3) $$G=\frac{\sum S}{A_{0}},$$

where ∑s is the sum of all of the aggregate fracture areas in the cross section and *A_0_* is the area of the cross section of the beam specimen, where *A_0_* = 35 × 30 = 1050 mm^2^, theoretically.

**Figure 13:**
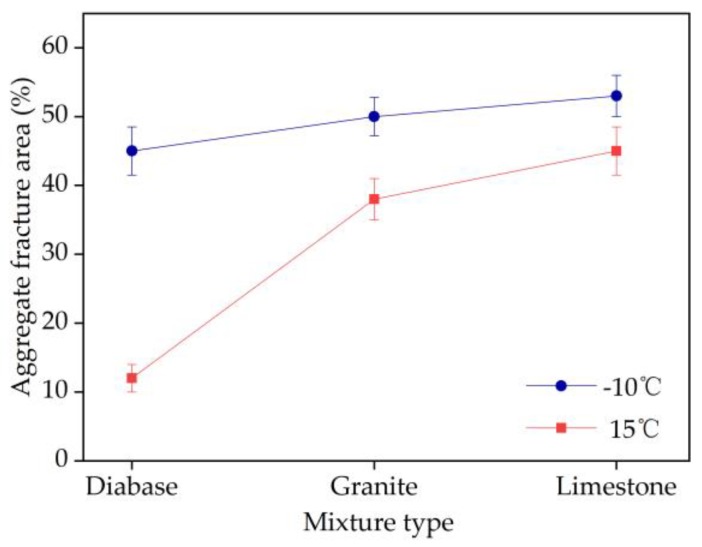
Aggregate fracture area ratio of specimen cross sections.

**Figure 14:**
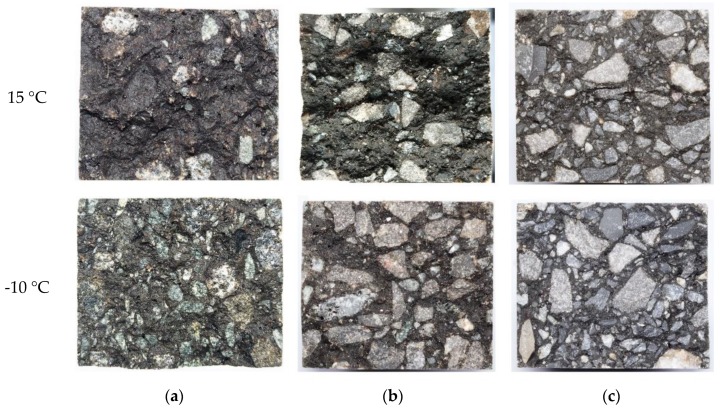
Fracture sections of beams of EACs containing: (**a**) diabase, (**b**) granite, and (**c**) limestone.

The statistical results of the aggregate fracture area ratio are shown in Figure 13. The cross sections of the EAC beam specimens of the different aggregate types are shown in Figure 14. At the same temperature, the aggregate fracture area of diabase EAC was smaller than those of the granite and limestone EACs. For example, at 15 °C, the aggregate fracture area ratios of the cross sections of the EAC specimens containing diabase, granite, and limestone were 12%, 38%, and 45%, respectively. At the test temperature of −10 °C, the proportion of fractured aggregates in the EACs increased, and the aggregate fracture area ratios of the cross sections of the EAC specimens containing diabase, granite, and limestone increased by 275%, 32%, and 18%, respectively. The analyses discussed in Section 4.3 showed that under the same conditions, the EAC flexural tensile resistance increased with the aggregate strength. Combining this result with the fracture analysis of aggregates of different strengths shows that the lower the ratio of fractured aggregates in an EAC, the higher the flexural tensile strength of the mixture. Thus, using high-strength aggregates to reduce the ratio of fractured aggregates significantly enhances the EAC flexural resistance.

### 4.7. Mixture Fracture Path Analysis

**Figure 15:**
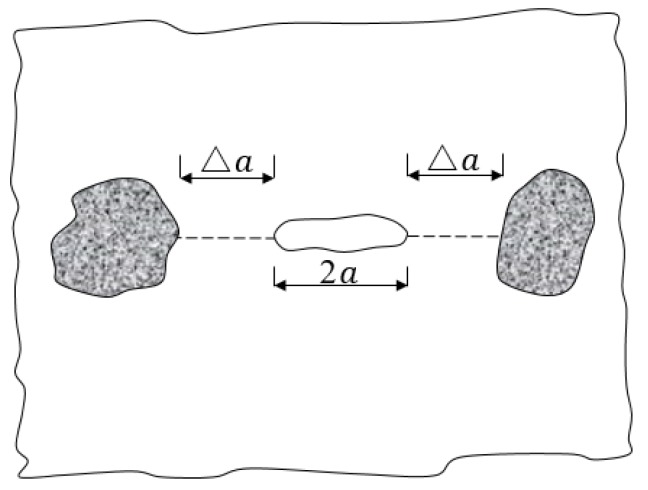
Schematic of a crack encountering an aggregate after propagating a distance Δa.

Energy is converted in the material failure process. Most of the strain energy accumulated in the material under loading is released in the form of fracture surface energy. EAC exhibits approximately linear elastic material characteristics under loading within a certain range of temperatures, and the Griffith formula can be used to describe crack propagation in the mixture [28]. A crack encounters aggregates while propagating in asphalt mastic, as shown in Figure 15. Assuming an initial crack length in asphalt mastic of *2*a, a crack propagates rapidly after the stress reaches$\;\sigma_{m}$, which is defined using the Griffith formula as follows:

(4) $$\sigma_{m}=\sqrt{\frac{2E\gamma_{m}}{\pi a_{m}}},$$

where $\gamma_{m}$ is the surface fracture energy of the asphalt mastic; $a_{m}\;$is the half-length of the critical crack; and $\sigma_{m}$ is the maximum critical stress required for the propagation of the crack front in the asphalt mastic.

The crack encounters the aggregate after propagating a distance of Δa. The condition for further crack propagation becomes

(5) $$\sigma_{j}=\sqrt{\frac{2E\gamma_{j}}{\pi (a_{m}+\Delta a)}},$$

where $\gamma_{j}$ is the fracture surface energy of the aggregate and $\sigma_{j}$ is the maximum critical stress required for the crack to pass through the aggregate.

In general, the fracture surface energy of the aggregate is greater than that of the asphalt mastic (i.e., $\gamma_{j}$ > $\gamma_{m})$. In fracture mechanics theory, a crack always propagates along the path of least energy dissipation [29]. Under normal circumstances, less energy is required for a crack to pass around than through an aggregate. The crack has already passed around an aggregate before the load is applied for the stress to reach $\sigma_{j}$.

**Figure 16:**
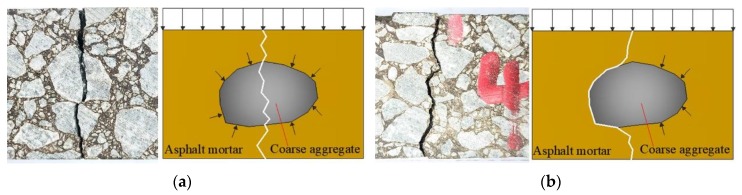
Fracture propagation by the crack path: (**a**) through an aggregate particle and (**b**) around an aggregate particle.

An epoxy asphalt mixture has high strength compared to ordinary asphalt concrete and significantly different fracture characteristics [30,31]. In an ordinary asphalt mixture, cracks usually propagate by passing around aggregates, and fracture failure mainly occurs at the bonding interface between the coarse aggregates and asphalt mastic. As aggregate fractures rarely occur, the interfacial bond strength is the dominant factor in the fracture of an ordinary asphalt mixture. In comparison, the EAC strength increased remarkably with the cohesion of the epoxy asphalt. Thus, the bond strength at the interface between the aggregates and the mastics increased. Griffith theory was used to compare the energy required for a crack to pass around versus through aggregates in an asphalt mixture, where the crack propagation direction corresponds to the lower energy path. These two crack propagation paths are shown in Figure 16. Even with significant strengthening of the interface between the epoxy asphalt mastic and the aggregate, some cracks still propagated inside the mixture by passing around the aggregates, showing that the interfacial bond strength between the aggregates and the mastic remains an important factor in EAC fracturing. The aforementioned bending tests showed that decreasing the temperature resulted in more aggregate fractures inside the mixture under loading. The viscoelastic behavior of EAC as a composite material made of epoxy asphalt and aggregate results in different responses under variable temperatures and loading rates [32]. When the temperature increases and the loading rate decreases, the viscosity of the mixture increases and the elasticity decreases. For low temperatures or higher loading rates, the epoxy asphalt exhibits more elastic and brittle behavior, which increases the stiffness of the subsequent epoxy asphalt mixture. At low temperatures, the strength of the epoxy asphalt increases, strengthening the interfacial bonding between the aggregate and the mastic. Thus, more energy is required for the crack to pass around than through the aggregate, and the crack propagates via the latter path. The fracture characteristics of EACs can be significantly improved by increasing the aggregate fracture strength. In summary, the aggregate strength affects the mixture resistance to flexural cracking when the binder strength and the interface cohesive strength in the mixture are similar to the aggregate strength.

**Figure 17:**
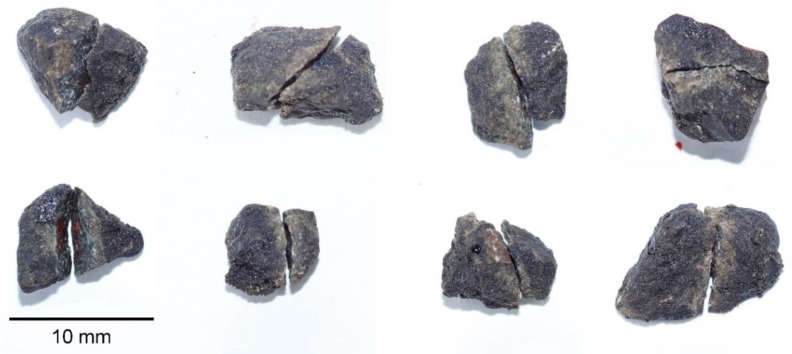
Aggregate fracture location distribution.

Studies have shown that coarse aggregate particles in an asphalt mixture will move (migrate and rotate) during the compaction process, and elongated, flat aggregate particles tend to eventually become horizontal [33,34]. From the torque condition for an object under force, elongated, flat aggregate particles are more likely to be broken off. A portion of the fractured aggregates at the fracture section of the mixture beam specimen were removed for observation, as shown in Figure 17. Most of the cracks passed through the middle part of the aggregates, resulting in an aggregate fracture, which is more likely to occur in elongated, flat aggregates. Thus, to reduce aggregate fracturing and thus improve the mixture crack resistance, it is recommended that cubic high-strength aggregates be used in EACs and that the content of flat and elongated particles be minimized.

### 4.8. Fatigue Property of the EAC Analysis

**Figure 18:**
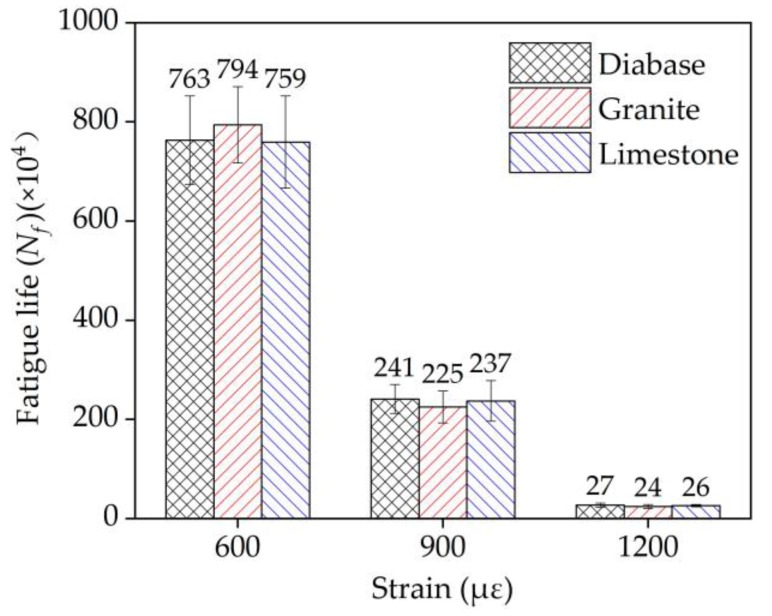
Fatigue test results of the three types of EACs.

**Figure 19:**
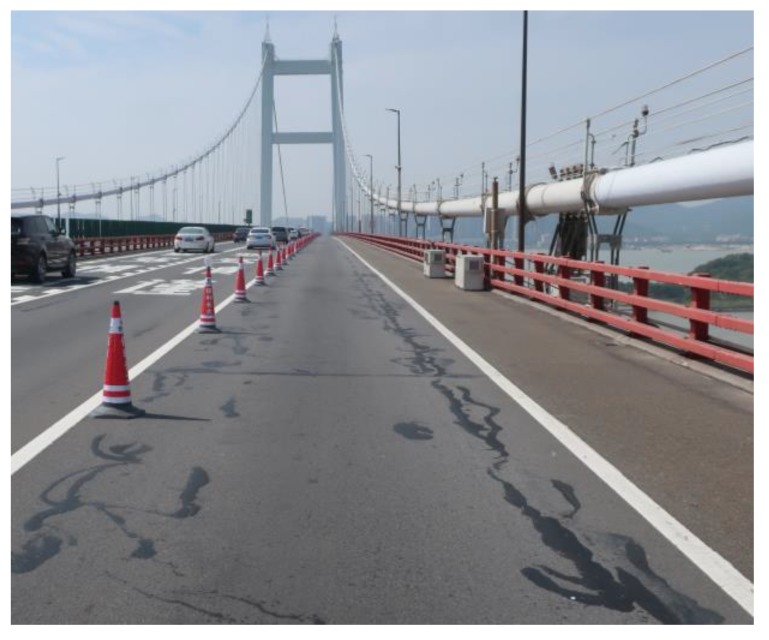
Photograph of fatigue cracking in the steel bridge deck EAC pavement.

The fatigue test results at strain-controlled levels of 600 με, 900 με, and 1200 με at 15 °C in Figure 18 show that the aggregate strength had no evident influence on the fatigue properties of EAC. The cause of this phenomenon was analyzed as follows: at these strain levels, the epoxy asphalt mastic exerts its main resistance to fatigue damage, and the strain level does not reach the point at which the strength of the aggregate has an effect. However, fatigue cracking does actually occur in epoxy asphalt mixture pavement, as shown in Figure 19. The actual fatigue cracking process in steel deck pavement is more complicated than the fatigue test due to the load action mode, temperature effect, etc. The effect of aggregate strength on the fatigue of EAC needs further research. The steel bridge deck pavement strain is generally 100–300 με under an axle load of 100 kN [35]. With reference to the fatigue test results and engineering application performance investigation of EAC on steel bridges for more than 12 years, the evaluated EAC showed good fatigue resistance.

**Figure 20:**
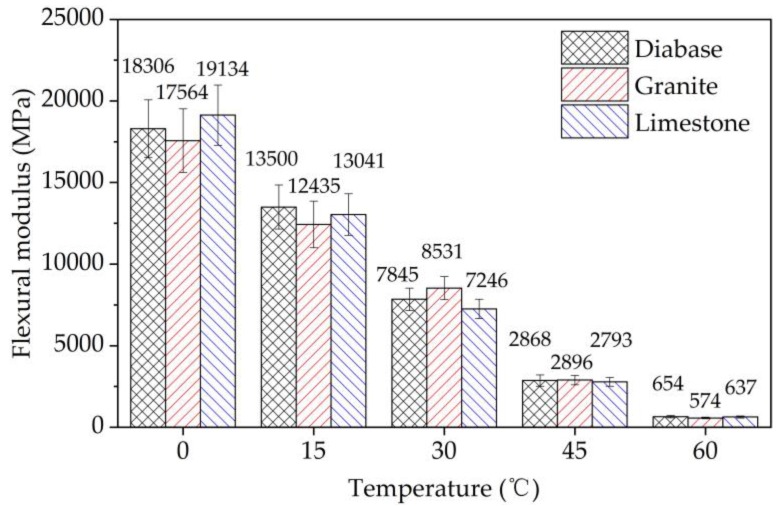
The initial flexural modulus in the four-point bending tests.

**Figure 21:**
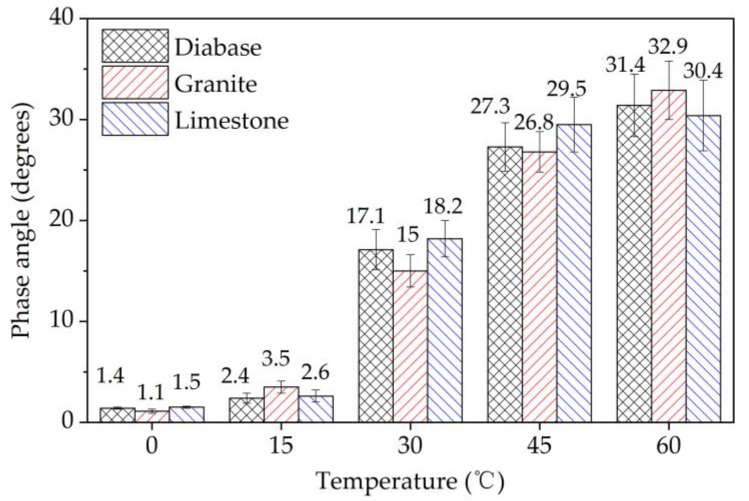
The initial phase angle in the four-point bending tests.

The initial flexural modulus and phase angle were obtained in the four-point bending tests to analyze the viscoelastic nature of the EACs, as shown in Figure 20 and Figure 21, respectively. The experimental data showed that as the test temperature increased, the modulus values of the EACs decreased, and the phase angle values of the EACs increased. This pattern also explains why when the temperature decreases, the effect of aggregate strength on the cracking performance of EAC gradually increases. Epoxy asphalt is the main factor influencing the viscoelasticity of EAC. The EACs showed elasticity below 15 °C and viscoelasticity above 30 °C. The aggregate strength had no evident influence on the initial flexural modulus or phase angle of the EACs, which was the same as the effect of different strength aggregate strengths on the fatigue performance of the EACs.

## 5. Conclusions

In this study, the influence of aggregate strength characteristics on EAC flexural cracking resistance was investigated through experiments.

(1) In beam bending tests, aggregate fractures in a general asphalt mixture were generally very rare, as fractures tended to develop between the EAC and the aggregate particles. Thus, the aggregate strength significantly influenced the EAC flexural cracking behavior. Bending tests are recommended to evaluate the effect of aggregates on EAC flexural cracking performance.
(2) The aggregate strength of EACs had no significant effect on the mixture’s Marshall stability in the Marshall tests, but had significant effects on the flexural tensile properties in the beam bending tests.
(3) The mastic in the EAC exhibited viscoelasticity, and the mechanical response of the mastic was much more sensitive to time and temperature than that of the coarse aggregates. The EAC flexural tensile strength was influenced by the interactions among the binder strength, the aggregate strength, and the test temperature. When the binder strength was close to the aggregate strength, the aggregate strength significantly impacted the flexural tensile properties of the mixture.
(4) Fatigue test results at strain-controlled levels of 600–1200 με and 15 °C showed that the aggregate strength had no evident influence on the fatigue properties of EAC. The effect of aggregate strength on EAC fatigue needs further research.

## Figures and Tables

**Table 1:** A-70 base asphalt (virgin) performance parameters.

Technical Indexes	Measured Value	Test Method
Penetration (25 °C, 0.1 mm)	63	ASTM D5
Softening point (°C)	49	ASTM D2398
Ductility (15 °C, cm)	>100	ASTM D113
Density (g/cm^3^)	1.036	ASTM D1298
Solubility (%)	99.7	ASTM D2042
Flash point (°C)	334	ASTM D92

**Table 2:** Performance parameters of epoxy asphalt.

Technical Indexes	Measured Value	Test Method
Penetration (25 °C, 0.1 mm)	17	ASTM D5
Softening Point (°C)	>100	ASTM D2398
Tensile strength (23 °C, MPa)	2.90	ASTM D 638

**Table 3:** Performance parameters of SBS modified asphalt (virgin).

Technical Indexes	Measured Value	Test Method
Penetration (25 °C, 0.1 mm)	53	ASTM D5
Softening point (°C)	79	ASTM D2398
Ductility (5 °C, cm)	30	ASTM D113
Viscosity (135 °C, Pa.s)	2.19	ASTM D4402

**Table 4:** Technical indexes of the coarse aggregates.

Technical Indexes	Diabase	Granite	Limestone	Test Method
Compressive strength (MPa)	125	104.5	102.7	ASTM C942
Crushing value (%)	14.0	17.5	16.4	ASTM C942
Los Angeles abrasion loss (%)	16.8	18.2	17.9	ASTM C131
Water absorption rate (%)	0.95	1.08	0.81	ASTM C127
Apparent specific gravity (g/cm^3^)	2.864	2.751	2.638	ASTM C127
Percentage of flat and elongated particles (%)	7.8	7.2	8.1	ASTM D4791

**Table 5:** Chemical compositions (wt%) of the coarse aggregates.

Chemical Compositions	Diabase/%	Granite/%	Limestone/%
SiO_2_	46.3	76.72	1
TiO_2_	1.12	2.08	–
Al_2_O_3_	7.32	17.89	0.27
Fe_2_O_3_	18.27	1.87	0.27
CaCO_3_	10.71	0.91	95.57
MgO	11.39	0.02	0.06
Na_2_O	1.41	–	–
P_2_O_5_	1.11	–	–
MnO	1.33	0.02	0.06
SO_3_	1.04	0.49	2.77

Note: SiO_2_ = silicon dioxide; TiO_2_ = titanium oxide; Al_2_O_3_ = aluminum oxide; Fe_2_O_3_ = iron oxide; CaCO_3_ = calcium carbonate; MgO = magnesium oxide; Na_2_O = sodium oxide; P_2_O_5_ = phosphorus pentoxide; MnO = manganese (II) oxide; SO_3_ = sulfur trioxide.

**Table 6:** Technical indexes of limestone powder.

Technical Indexes	Criteria	Measured Value	Test Method
Water content (%)	≤1	0.30	JTG E42-2005 T0317
Hydrophilic coefficient	<1	0.48	JTG E42-2005 T0343
Plasticity index	<4	1.8	JTG E42-2005 T0354
Apparent specific density (g/cm^3^)	≥2.50	2.713	JTG E42-2005 T0352

**Table 7:** Gradation of the epoxy asphalt concrete (EAC) mixture (percentage by weight passing sieves).

**Screen Size (mm)**	13.2	9.5	4.75	2.36	1.18	0.6	0.3	0.15	0.075
**Percent Passing (%)**	100	99.0	74.5	55.6	42.6	35.4	24.6	18.1	11.7

**Table 8:** Marshall test results for the three EACs.

Aggregate Types	Air Void Content/%	Optimum Asphalt Content/%	Stability/kN	Flow Value/10^−1^ mm
Diabase	1.0	6.5	71.3	41.5
Granite	0.9	6.4	73.2	40.4
Limestone	1.1	6.2	72.4	43.2

**Table 9:** Three-point bending test results for the three types of aggregate asphalt mixtures.

Binder Types of Mixture	Test Temperature/°C	V_max_/mm	P_max_/N	Flexural Tensile Strength/MPa	Maximum Ultimate Flexural Tensile Strain/10^−6^
A-70	15	1.87	937.1	7.7	9818
SBS		2.03	1042.0	8.7	10658
Epoxy asphalt		2.26	3037.6	24.5	11863
A-70	−10	0.40	1194.4	9.8	2078
SBS		0.59	1418.6	11.6	3116
Epoxy asphalt		0.76	4618.4	37.3	4075

Note: *V_max_* is the maximum deflection in the span of the mixture beam and *P_max_* is the critical failure load of the mixture beam.

**Table 10:** Three-point bending test results for EACs with the three types of aggregates.

Aggregate Types	Specimen Number	15 °C		−10 °C	
		V_max_/mm	P_max_/N	V_max_/mm	P_max_/N
Diabase	1	2.15	3091.9	0.77	4688.4
	2	2.53	2948.1	0.88	4748.8
	3	2.09	3148.0	0.72	4522.2
	4	2.10	3064.2	0.72	4515.8
	5	2.37	3013.9	0.71	4645.3
	6	2.33	2959.8	0.76	4589.7
	Mean values	2.26	3037.6	0.76	4618.4
Granite	1	1.77	2899.1	0.60	3784.2
	2	1.58	2804.1	0.71	3896.4
	3	1.97	3054.5	0.65	3728.6
	4	1.70	2875.6	0.68	3852.1
	5	2.00	2967.6	0.70	3805.4
	6	1.63	3240.7	0.64	3778.9
	Mean values	1.77	2973.6	0.66	3807.6
Limestone	1	1.17	2988.0	0.69	3675.0
	2	1.28	3025.9	0.73	3652.1
	3	1.23	3144.0	0.67	3702.0
	4	1.12	3148.6	0.68	3728.6
	5	1.28	3036.1	0.71	3689.7
	6	1.17	3185.3	0.67	3654.5
	Mean values	1.21	3088.0	0.69	3683.7

Note: *V_max_* is the maximum deflection in the span of the mixture beam and *P_max_* is the critical failure load of the mixture beam.
